# Addition of Phenylboronic Acid to *Malus domestica* Pollen Tubes Alters Calcium Dynamics, Disrupts Actin Filaments and Affects Cell Wall Architecture

**DOI:** 10.1371/journal.pone.0149232

**Published:** 2016-02-17

**Authors:** Kefeng Fang, Sai Gao, Weiwei Zhang, Yu Xing, Qingqin Cao, Ling Qin

**Affiliations:** Beijing Key Laboratory for Agricultural Application and New Technique, College of Plant Science and Technology, Beijing University of Agriculture, Beijing 102206, China; Wuhan University, CHINA

## Abstract

A key role of boron in plants is to cross-link the cell wall pectic polysaccharide rhamnogalacturonan-II (RG-II) through borate diester linkages. Phenylboronic acid (PBA) can form the same reversible ester bonds but cannot cross-link two molecules, so can be used as an antagonist to study the function of boron. This study aimed to evaluate the effect of PBA on apple (*Malus domestica*) pollen tube growth and the underlying regulatory mechanism. We observed that PBA caused an inhibition of pollen germination, tube growth and led to pollen tube morphological abnormalities. Fluorescent labeling, coupled with a scanning ion-selective electrode technique, revealed that PBA induced an increase in extracellular Ca^2+^ influx, thereby elevating the cytosolic Ca^2+^ concentration [Ca^2+^]c and disrupting the [Ca^2+^]c gradient, which is critical for pollen tube growth. Moreover the organization of actin filaments was severely perturbed by the PBA treatment. Immunolocalization studies and fluorescent labeling, together with Fourier-transform infrared analysis (FTIR) suggested that PBA caused an increase in the abundance of callose, de-esterified pectins and arabinogalactan proteins (AGPs) at the tip. However, it had no effect on the deposition of the wall polymers cellulose. These effects are similar to those of boron deficiency in roots and other organs, indicating that PBA can induce boron deficiency symptoms. The results provide new insights into the roles of boron in pollen tube development, which likely include regulating [Ca^2+^]c and the formation of the actin cytoskeleton, in addition to the synthesis and assembly of cell wall components.

## Introduction

Boron is known to be essential for plant growth as it functions as a crosslinking molecule in the cell wall and participates in other processes, such as maintenance of plasma membrane function, and in several metabolic pathways [1–2]. Accordingly, 90% of cellular boron is localized in the cell wall [3]. Borate contains two pairs of hydroxyl moieties that can form reversible diester bonds with molecules containing *cis*-diols in a favorable conformation, so it has a cross-linking capacity [4]. The importance of borate as a cross-linking molecule in plants has been emphasized by the discovery of borate-associated molecules, notably the pectic plant cell wall polysaccharide rhamnogalacturonan II (RG-II) [4–5]. The cross-linking of RG-II by boron contributes to cell wall architecture and consequently the biomechanical properties of growing cells [6]. Phenylboronic acid (PBA) is structurally similar to borate and can form reversible ester bonds with *cis*-diols; however, PBA has only one pair of hydroxyl moieties and so cannot cross-link two discrete molecules [7]. Therefore, boron diester cross-links can disrupted by the addition of PBA, which forms monoester linkages at positions normally occupied by boron. As a result, the addition of PBA to plants organs can induce a boron deficiency-like response, thereby providing a useful tool for investigating boron function in plants [4]. In addition, fluorescein boronic acid conjugates can been used as markers for borate-binding sites [8].

Pollen tubes, which grow over long distances to transport the male gametes to the embryo sac for fertilization, are an example of a rapidly growing cell type that can be used to study cell expansion under semi-*in vitro* conditions. They elongate by tip growth, meaning that their elongation exhibits a polarized growth pattern where secretory vesicles derived from the Golgi apparatus transport large amounts of membrane and cell wall precursors to the elongating cell tip [9]. The secretory vesicle membranes fuse with the plasma membrane, the released precursors are incorporated into the new cell wall at the pollen tube tip, enabling cell elongation [10]. It is generally accepted that the wall of the pollen tube differs from that of other plant cells, in that it consists of a primary pecto-cellulosic layer and a secondary callosic wall [11]. The callosic inner lining is absent in the tip cell wall, and so pectins appear to be the major component of the cell wall in this region [11–12]. Previous studies have shown that highly esterified pectins are localized in the tip of the growing pollen tube, while de-esterified pectins occupy areas outside this zone [11]. De-esterified pectins are cross-linked by Ca^2+^ ions to form a semi-rigid pectate gel, thereby altering the physical properties of the wall and providing mechanical support for the distal areas of the elongating pollen tube [13]. The esterified pectins that are present predominantly at the apex are thought to be important in allowing turgor-driven directional expansion of the tube [14]. The cross-linking of RG-II by boron has been proposed to contribute to cell wall architecture, and to influence the mechanical properties of growing cell walls [6, 15]. Consistent with this idea, a gene that is required for borate cross-linking of RG-II is necessary for plant reproductive tissue development and fertilization [16]. Mutations that alter RG-II structure, thereby decreasing its ability to form cross-links with borate, have also been shown to be deleterious to plant growth and development [17]. So both de-esterified and esterified pectins contribute jointly to the growth of plant cell [18].

Pollen tubes are rapidly growing and require a high concentration of boron to germinate and maintain cell elongation [19], making them a good system for studying the competitiveness of PBA for boron-binding sites. They are sensitive to boron deficiency, and the morphological effects of boron deficiency on pollen tube growth have been investigated [20]. Given apple is the fruit tree species that is most affected by boron deficiency [21], which is a major agriculture problem that causes large losses in yield [1]. In this current study, apple (*Malus domestica*) pollen tubes were chosen as a model for studying the effects of PBA on tube polar growth to decipher the mechanism underlying.

## Results

### PBA affected pollen germination and tube growth

As we mentioned in introduction, addition of PBA could disrupt boron diester cross-links by forming monoester linkages at positions normally occupied by boron. So, the addition of PBA to plants organs can induce a boron deficiency-like response. In the present study, different concentrations of PBA were used in the culture medium to observe its effect on apple pollen germination and tube growth and possible mechanism. No obvious difference in germination rate was observed when exogenous 0.01% H_3_BO_3_ was applied, indicating that the endogenous boron concentration was sufficient for pollen germination; however, exogenous boron promoted tube growth leading to a much longer tube. PBA inhibited pollen germination and tube growth in a dose-dependent manner (Table 1, S1 Fig). Based on the results presented in Table 1, 0.5 mM PBA was used in the subsequent experiments.

**Table 1:** Effect of H_3_BO_3_ and PBA on pollen germination and tube growth.

Concentration	Germination rate/%	Tube length/μm
0	36.33 ± 2.19a	132.51 ± 1.12d
0.01% H_3_BO_3_	38.75 ± 1.13a	219.66 ± 6.22a
0.1 mM PBA	26.18 ± 2.98b	175.15 ± 5.12b
0.3 mM PBA	23.36 ± 0.79bc	163.43 ± 0.87c
0.5 mM PBA	17.49 ± 2.42cd	104.06 ± 5.92e
0.6 mM PBA	7.37 ± 1.13e	69.15 ± 9.15f
0.7 mM PBA	3.44 ± 0.91e	58.54 ± 1.33f

Pollen germination rate and tube length were calculated after 2h culture in different media. Different letters indicate statistically significant differences between pollen tubes grown in various concentrations of PBA compared to the control samples, as determined by Student’s t test (P < 0.05); n = 150 from three independent experiments.

**Fig 1:**
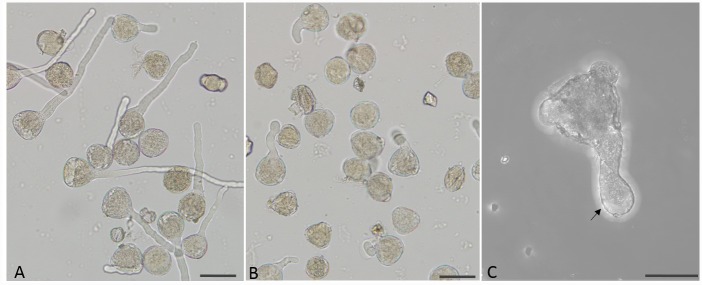
Morphology of pollen tubes. (A) Control pollen tubes. (B) Pollen tubes treated with 0.5 mM PBA. (C) PBA treated abnormal pollen tube showing a swollen tip (arrow). Scale bars = 50μm.

In germination medium composed by 20% (w/v) sucrose and 0.01% CaCl_2_, pollen tubes seemed healthy and had a constant diameter as shown in Fig 1A, whereas the morphology of pollen tubes treated with 0.5 mM PBA for 2 h was abnormal with swollen tip (Fig 1B and 1C).

### PBA induced an increase in extracellular Ca^2+^ influx and an elevation in [Ca^2+^]c, as well as the disappearance of the [Ca^2+^]c gradient in the pollen tube tip

**Fig 2:**
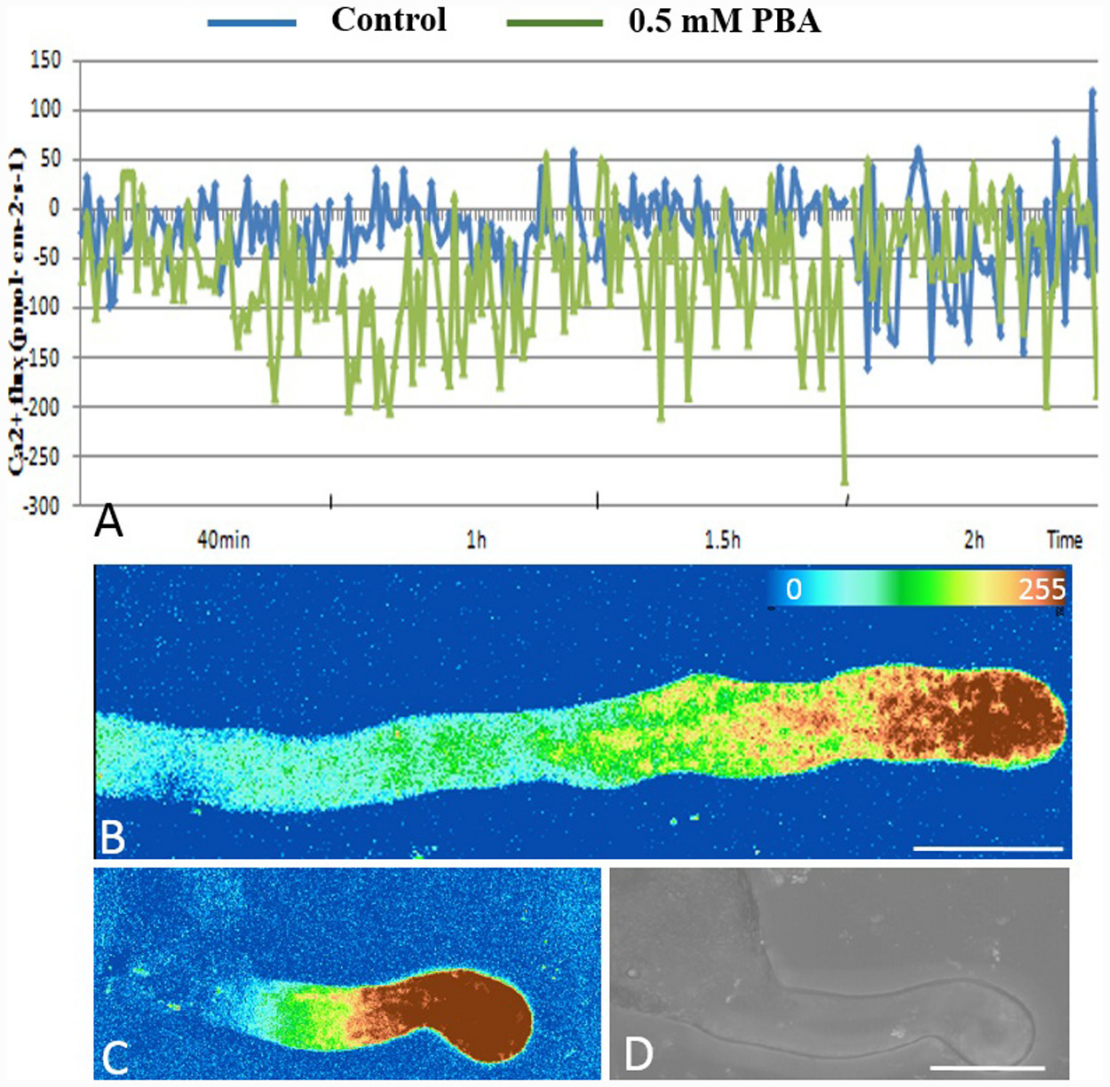
Influx of calcium at the apex of the pollen tube and [Ca^2+^]c. (A) Influx of calcium at the apex of the pollen tube at different time points. The blue line represents the control, while the green line represents pollen tube treated with PBA. (B) The [Ca^2+^]c gradient at the apex of the control pollen tube. (C) Strong fluorescence was detected at the apex of the pollen tube treated with PBA, indicating the disappearance of the [Ca^2+^]c gradient. (D) Bright field image of C. Scale bars = 25μm

We measured Ca^2+^ influx at the extreme apex of growing pollen tubes using a vibrating electrode technique. We found Ca^2+^ influx in the control tube apex at all the time points, with a mean maximal Ca^2+^ influx at the peak of the oscillation of 160.34 pmol cm^-2^ s^-1^ (±7.84, n = 5; Fig 2A). PBA increased markedly the magnitude of the Ca^2+^ influx at the extreme apex with the maximal influx 264.45 pmol cm^-2^ s^-1^ (±6.49, n = 5), indicating that the net cytosolic Ca^2+^ concentration ([Ca^2+^]c) derived from extracellular Ca^2+^ was substantially increased by the application of PBA.

We also visualized [Ca^2+^]c using Fluo-3/AM dye. Control pollen tubes displayed a representative tip-focused [Ca^2+^]c gradient at the tip region (Fig 2B), while the pollen tubes treated with 0.5 mM PBA showed much stronger [Ca^2+^]c fluorescence in their swollen tips. Interestingly, this [Ca^2+^]c gradient was absent in the tip of PBA treated tube (Fig 2C) indicating that PBA promoted an accumulation of [Ca^2+^]c and a disappearance of the [Ca^2+^]c gradient in the tip.

### PBA treatment resulted in the distortion of actin filaments

**Fig 3:**
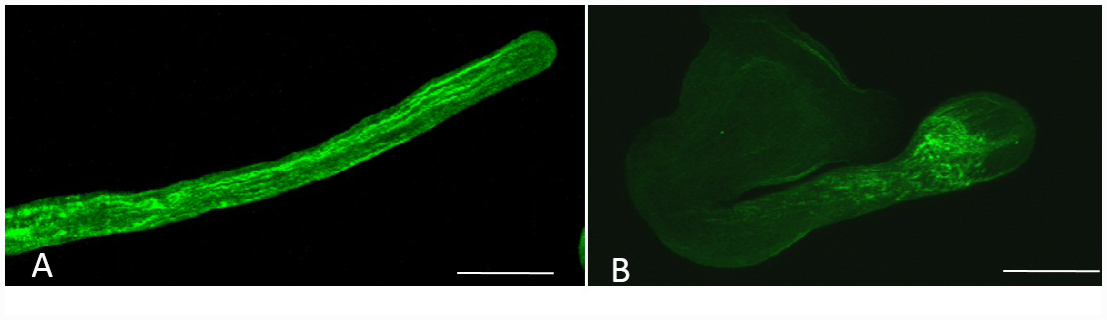
Actin filaments in pollen tubes grown in different media. (A) Parallel actin filaments in a pollen tube in normal culture medium. Scale bars = 25μm. (B) Disrupted actin filament distribution in the subapical region of the pollen tube treated with 0.5 mM PBA. Scale bar = 25μm.

Actin filaments is involved in vesicle trafficking, plant cell wall formation and pollen tube tip growth [11, 22], so the actin cytoskeleton of control and PBA-treated pollen tubes was compared. Using LSCM, the actin filaments of the control pollen tubes were shown to be a network of cables in the whole tube except the tip which parallel to the growth axis (Fig 3A). However, PBA induced the actin filaments to be twisted and condensed, and the disrupted actin filament fragments accumulated in clusters in the subapical region (Fig 3B).

### Influence of PBA on pectin and AGP deposition

**Fig 4:**
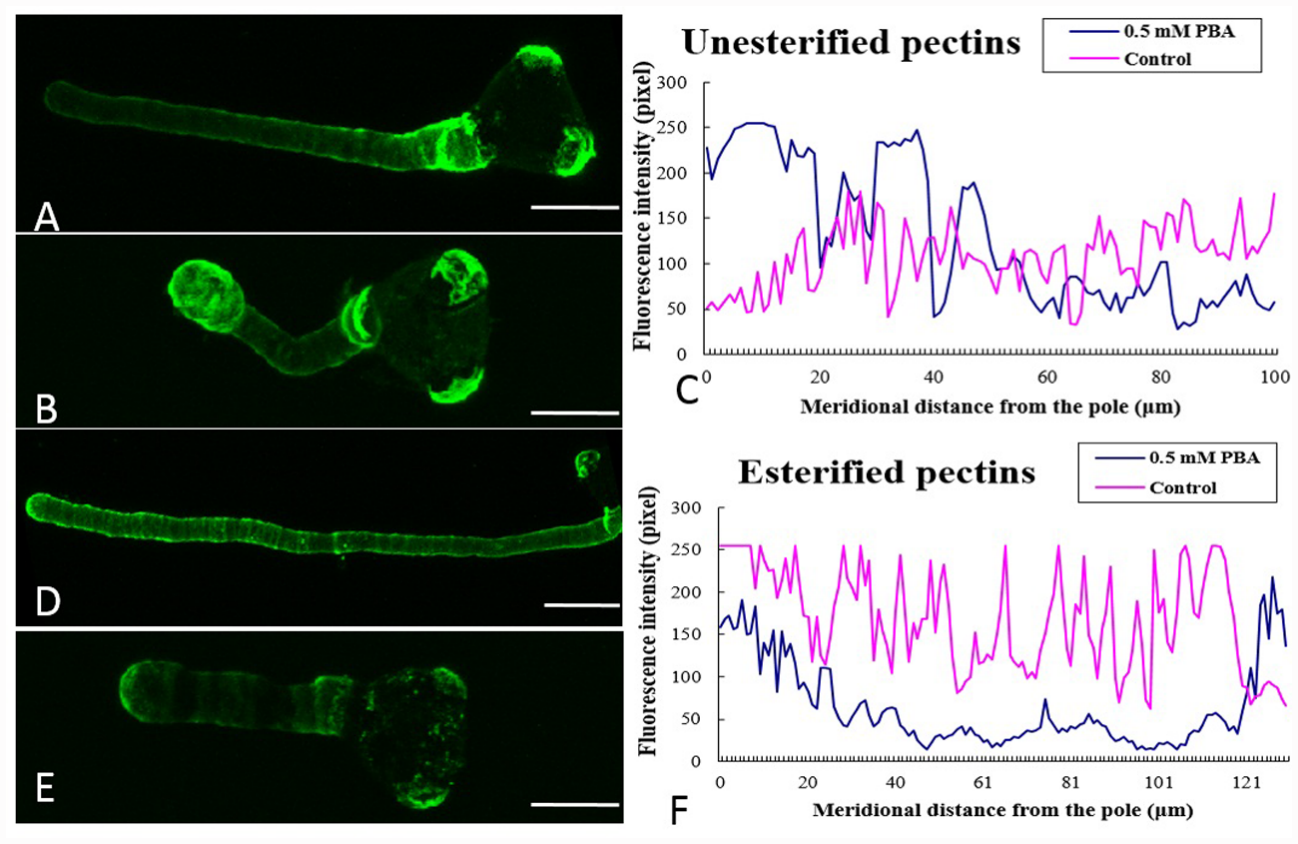
Influence of PBA on distribution of de-esterified (JIM5) and esterified pectins (JIM7). (A) More de-esterified pectins indicated by JIM5 fluorescence were present at the basal part of the pollen tube in normal medium, whereas less in the apical region. (B) The signal intensity of JIM5 labeling indicated that there was more de-esterified pectin present in the apex of the PBA treated pollen tubes. (C) Quantitative analysis of the fluorescence signal obtained after JIM5 labeling of the wall of control pollen tubes (control, pink line) and PBA-treated tubes (0.5 mM PBA, blue line), indicating that PBA promoted the accumulation of de-esterified pectin at the apex of the tubes. (D) Higher levels of esterified pectins were present in the apical region of pollen tubes cultured in normal medium, indicated by fluorescence signal of JIM7. (E) Fluorescence was observed mainly at the tube tip and some at the base after JIM7 labeling of pollen tubes in the presence of PBA. (F) Quantitative analysis of the fluorescence signal obtained after labeling the wall of control pollen tubes (control, pink line) and PBA-treated tubes (0.5 mM PBA, blue line) with JIM7, showing that PBA reduced the accumulation of esterified pectin at the tube. Scale bars = 25μm.

In control pollen tubes, the distribution of JIM5 labeled (de-esterified) pectins was relatively uniform, with a higher concentration at the basal region near the grain and less at the tip (Fig 4A and 4C). In contrast, in pollen tubes treated with 0.5 mM PBA, higher levels of de-esterified pectins were detected across the tip surface and cytoplasm, as well as in the basal region near the grain, whereas levels were lower on the shank of the tubes (Fig 4B and 4C). In control pollen tubes, the distribution of JIM7 labeled (esterified) pectins was relatively uniform along the tube, but with stronger fluorescence at the tip of the growing tubes (Fig 4D and 4F), while esterified pectins were detected at the tip of PBA treated tubes, including the basal areas (Fig 4E and 4F). We hypothesized that the larger amounts of de-esterified pectin in the pollen tubes treated with PBA inhibited their growth, and quantitative analysis confirmed that the addition of PBA changed the distribution and fluorescence intensity of both de-esterified and esterified pectins (Fig 4C and 4F).

**Fig 5:**
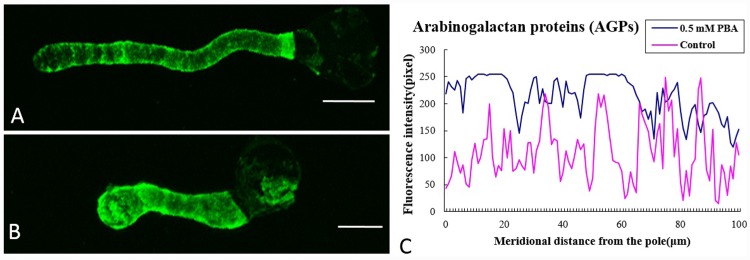
Influence of PBA on the distribution of arabinogalactan proteins (AGPs). (A) Fluorescence after labeling pollen tubes cultured in normal medium with the LM2 antibody, indicating a greater abundance of AGPs in the basal region and decreased levels from the base towards the pollen tip. (B) Pollen tubes treated with PBA, showing fluorescence of LM2 throughout the tube, with a higher signal at the tip and adjacent to the basal region near the grain. (C) Quantitative analysis of the fluorescent signal obtained after labeling the wall of control pollen tubes (control, pink line) and PBA-treated tubes (0.5 mM PBA, blue line) with LM2, indicating that PBA induced accumulation of AGPs at the pollen apex. Scale bars = 25μm.

Arabinogalactan proteins (AGPs) are complex proteoglycans of the cell wall found in the entire plant kingdom and in almost all plant organs and involved in a large number of biological functions including reproduction [23]. mAbs LM2 could recognize and bind to ß-linked GalA epitopeof AGPs, and were used to label AGPs in rice and carrots [23]. In the present study, LM2 was chosen to label AGPs. The PBA treatment also caused AGPs to accumulate throughout the pollen tube, except in the basal region near the grain (Fig 5B), which contrasted with the characteristic periodic ring-like deposits and less signal at the tip in the control cells (Fig 5A and 5C).

### Effect of PBA on cellulose and callose deposition

**Fig 6:**
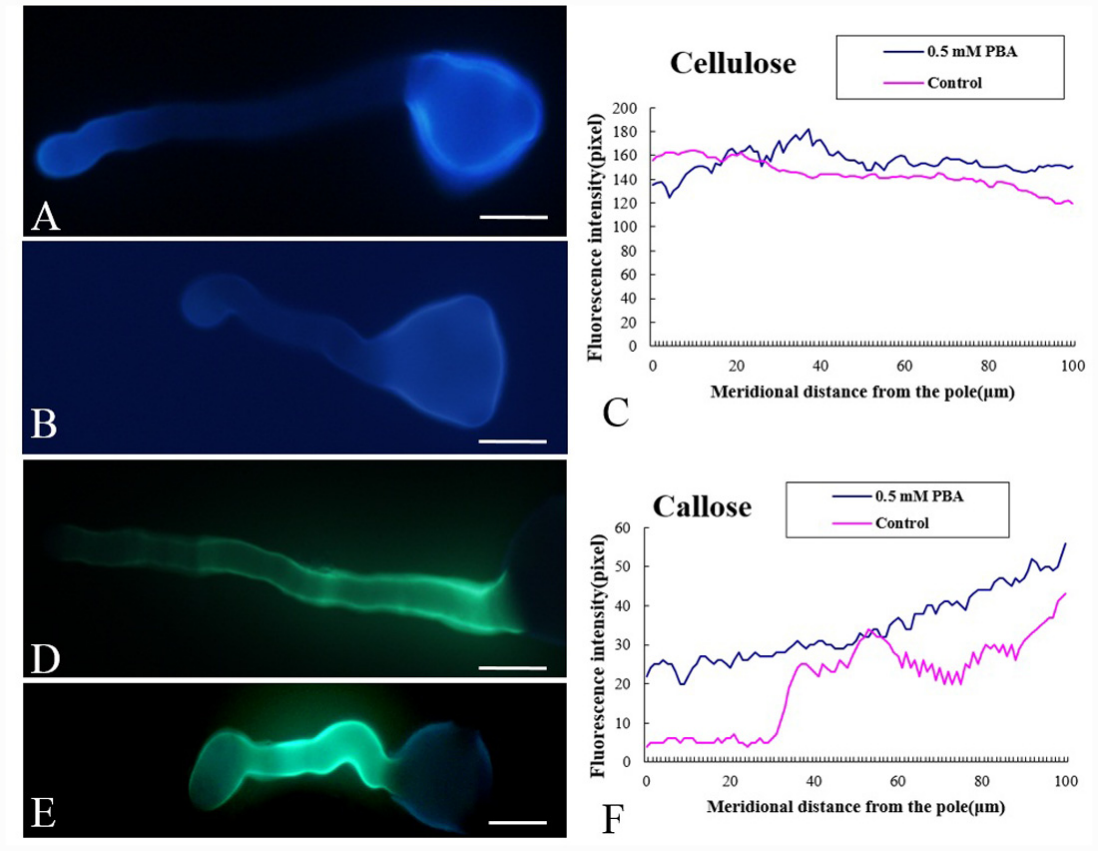
Effect of PBA on the distribution of cellulose and callose in pollen tube. Scale bars = 25μm. (A) Fluorescence of calcofluor white showed that cellulose distributed along the whole pollen tube. (B) PBA treated pollen tube were labeled by Calcofluor, showing that cellulose was present on the whole tube. (C) Quantitative analysis of the fluorescent signal from Calcofluor white staining of cellulose in the wall of control pollen tubes (control, pink line) and PBA treated tubes (0.5 mM PBA, blue line). (D) Callose distribution along the control pollen tube except for at the tip indicated by fluorescent signal from aniline blue. (E) Distribution of callose along the whole PBA treated pollen tube, with weak signal at the tip. (F) Quantitative analysis of the fluorescent signal from aniline blue staining of callose in the wall of control pollen tubes (control, pink line) and PBA treated tubes (0.5 mM PBA, blue line).

Cellulose, detected using Calcofluor white, was relatively uniformly distributed in both the control (Fig 6A) and PBA treated pollen tubes (Fig 6B), with no obvious differences between the tubes, indicating that PBA had no effect on cellulose deposition. This conclusion was further supported by quantitative analysis (Fig 6C and 6D). Callose, visualized by aniline blue staining, was distributed fairly evenly along the tube shank but was not present at the tip in the control tubes (Fig 6D and 6F), whereas callose was present at the tip of the PBA treated pollen tubes, as well being present along the shank (Fig 6E and 6F).

### FTIR spectroscopy analysis of pollen tube wall components

**Fig 7:**
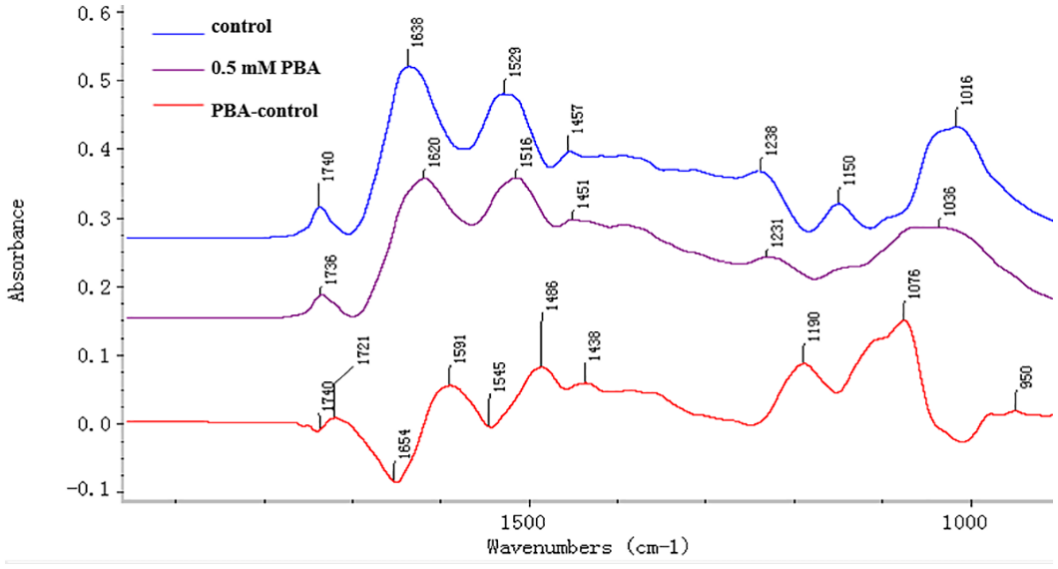
FTIR spectra gained from analysis of the tip regions of normal pollen tubes (control, blue line), pollen tubes treated with 0.5 mM phenylboronic acid (PBA, pink line), and the FTIR differential spectrum generated by digital subtraction of the control spectra from the spectra of the PBA treated samples (PBA-control, red line).

Pollen tubes were imaged by FTIR spectroscopy to compare the abundance of chemical bonds/ moieties that can be associated with different cell wall components. Fig 7 displayed representative FTIR spectra obtained from the tip region of control and 0.5 mM PBA treated pollen tubes. In the control pollen sample, saturated esters, protein, carboxylic acid groups and carbohydrates absorbed at 1740 cm^-1^, 1638 and 1529 cm^-1^, 1457 cm^-1^, and between 1200 and 900 cm^-1^ respectively. PBA induced displacements and changes in the absorbance such that the ester peak decreased, while the free acid stretches increased proportionally and the peaks of protein amide stretching bonds were displaced to 1620 and 1516 cm^-1^, respectively. The FTIR differential spectrum showed that the content of proteins (amide I absorbed at 1591 cm^-1^, amide II at 1529 and 1486 cm^-1^) and de-esterified pectin (absorbed at 1457 and 1438 cm^-1^) increased, while the content of esterified pectin (absorbed at 1740 cm^-1^) and protein amide I (absorbed at 1654 cm^-1^) decreased slightly after the PBA treatment, which supports the results of the fluorescence labeling.

## Discussion

Boron is known to affect mechanical properties of the plant cell wall and also to cross-link glycosylinositol phosphorylceramides in the plasma membrane with AGPs in the cell wall, thereby attaching the membrane to the wall [2,24]. Apple pollen grains have been reported to contain 55.45 μg/g boron [25], and our results showed that the endogenous levels were able to support pollen germination, but were not sufficient for subsequent tube growth. Experiments with germinating petunia (*Petunia hybrida*) pollen and boronate affinity chromatography have previously shown that PBA and boron compete for the same binding sites [4]. PBA has been shown to specifically disrupt or prevent borate dependent crosslinks that are important for the structural integrity of the cell, including the organization of transvacuolar cytoplasmic strands of tobacco cells, making it a useful tool for rapidly and reversibly inducing boron deficiency-like responses [4].

### Effect of PBA on pollen germination and tube growth

Boron is an essential microelement that is required for the growth and development of plants [1]. We found that PBA treatments reduced the pollen germination rate, leading to a retardation of pollen tube growth, which has been noted as a boron deficiency symptom [20]. In the present study, pollen tubes cultured in control germination medium had a normal shape and length; however, when PBA was added to the medium at the beginning of the culture period, pollen germination and tube growth were inhibited and pollen tube morphological abnormalities were observed. This occurred in a dose-depended manner since with increasing PBA concentrations, the frequency of pollen tube abnormalities increased and the average pollen tube growth rate decreased. We propose that the pollen tube wall assembly and architecture was perturbed by the PBA treatment conditions, which affected the biomechanical properties of the tube wall.

### PBA induced extracellular calcium influx and disappearance of the [Ca^2+^]c gradient

It has been reported that calcium, a universal secondary messenger, mediates the coupling of stimulus-triggered responses to regulate diverse cellular functions during pollen tube development [26] and, as such, calcium influx and the tip-focused calcium gradient are indispensable. A tip-to-base cytoplasmic calcium ([Ca^2+^]c) gradient was identified in growing pollen tubes of *Pinus bungeana*, and this was largely maintained by extracellular Ca^2+^ influx and considered to be important for the regulation of cellular growth and orientation [27]. Short-term boron deficiency in tobacco BY-2 cells expressing the calcium binding protein aequorin increases Ca^2+^ uptake [28]. In addition, it appears that boron and Ca^2+^ affect gene expression in both BY-2 cells and legume nodules [29–30]. Boron deficiency also increases the cytosolic Ca^2+^ levels and the expression of genes involved in Ca^2+^ signaling in *Arabidopsis thaliana* roots [31]. González-Fontes et al. [31] proposed a hypothetical mechanism based on these observations, assigning Ca^2+^ a key role in the mechanism through which plants may sense and transduce the boron deprivation signal and modulate physiological responses.

In the present study, PBA was shown to promote extracellular Ca^2+^ influx and increase the tip-focused [Ca^2+^]c concentration as well causing the disappearance of the [Ca^2+^]c gradient. There is a close association between the intracellular tip-focused [Ca^2+^]c gradient, the extracellular tip directed Ca^2+^ influx, and elongation of the pollen tube [27]. On the basis of our results related to the [Ca^2+^]c gradient and Ca^2+^ influx, we conclude that boron regulates the [Ca^2+^]c gradient largely by mediating the Ca^2+^ influx, thereby regulating pollen tube development. Another explanation for the increased concentration of Ca^2+^ in the apex following the PBA treatment, is that PBA form bonds with *cis*-diols but does not produce binding sites for calcium ions, resulting in higher [Ca^2+^]c concentrations.

### PBA disturbed the organization of actin filaments

There is considerable evidence that actin filaments control cytoplasmic streaming and hence the transport of secretory vesicles, and that actin polymerization itself also contributes to pollen tube growth and cell shape [11, 32–34]. In the elongating pollen tubes, actin filaments are arrayed in bundles that extend along the longitudinal axis, reaching the subapical region [11, 34]. There is crosstalk between Ca^2+^ signaling and the cytoskeleton in the pollen tube, and the configuration of the actin filaments is controlled via numerous regulatory factors, including several actin-binding proteins, most of which are regulated by the Ca^2+^ signal [35]. Ca^2+^ is a central factor controlling the transition from the G-actin present in the tube apex to the F-actin cables in the shank [36]. Short and long-term boron deficiency changed cytoskeleton biosynthesis, suggesting the existence of a direct interaction of boron with the cytoskeleton [21, 37]. In this current study, the organization of actin filaments in pollen tubes was disrupted when they were treated with PBA, indicating that there is a close relationship between the actin cytoskeleton and boron during pollen tube elongation. We speculate that the sensitivity of F-actin organization in the tip region of the pollen tubes to boron deficiency might be partly dependent on the Ca^2+^ gradient during boron signaling. We observed that PBA induced extracellular Ca^2+^ influx and resulted in an increased [Ca^2+^]c and the disappearance of the [Ca^2+^]c gradient, which we hypothesize caused actin de-polymerization.

### PBA treatment altered pollen tube cell wall composition

Since PBA affected the morphology of the pollen tubes, we investigated whether the chemical composition of the tube wall was affected. Calcofluor labeling showed that cellulose was present throughout the control pollen tube wall, including the tube tip. In the presence of PBA, the fluorescent signal intensity showed no obvious change, indicating that PBA does not inhibit the synthesis and deposition of cellulose in the pollen tube. Similarly, the PBA treatment caused no apparent changes in the abundance of distribution of callose, another major polysaccharide component in the pollen tube wall [38].

In addition to cellulose and callose, pollen tube walls contain a pectin network [11]. The cross-linking of the pectic polysaccharide components by boron has been proposed to make an important contribution to cell wall architecture, and therefore influence the mechanical properties of growing cell walls [14]. Our immunolabeling analysis showed that de-esterified pectin was mainly distributed on the flanks of the pollen tube, and that in PBA treated pollen tubes, it was present primarily in the apical and basal regions of the pollen tube, but was absent from the flank. Under normal conditions, de-esterified pectin can be cross-linked by Ca^2+^, providing mechanical strength to the tip wall; however, in the PBA-treated pollen tubes, most of de-esterified pectins accumulated at the tip, thereby reducing the normal degree of wall stiffening in the shank. Immunolabeling with JIM7 detected esterified pectin at the tips of normal pollen tubes, and there was minor decrease in the distribution of esterified pectin after the PBA treatment, indicating that PBA decreased the esterified pectin in the pollen tubes.

Bassil et al. [4] indicated that the effect of boron on cytoskeletal function could be either direct, by affecting the organization or stability of cytoskeletal components, or indirect, through its interaction with other cytoskeletal binding or anchoring molecules, such as glycoproteins and/or glycolipids. One such group of anchoring molecules are the glycosylphosphatidy linositol-anchored AGPs, which exist on the apoplastic side of the plasma membrane and can interact with pectins or other cell wall-localized proteins [39]. Recent studies have shown that periplasmic AGPs can bind Ca^2+^ through carboxyl groups on glucuronic acid residues, which act as potential intramolecular Ca^2+^-binding sites [40–41]. AGPs may participate in the boron deficiency signal transduction by binding Ca^2+^, while González-Fontes et al. [31] proposed that AGPs may be key components in Ca^2+^ signaling pathways, or participate in the anchoring of AGPs to the plasma membrane through the stabilization of their glycosylphosphatidy linositol lipid anchor. In the present study, we observed that the PBA treatment caused AGPs to accumulate along the whole pollen tube, but not in the basal region near the grain. This differed from the characteristic periodic ring-like deposits and lower levels at the tip that were seen in un-treated tubes. Thus, PBA changed the distribution pattern and quantity of AGPs, which we suggest may be linked to alterations in Ca^2+^ concentrations and actin organization.

FTIR spectroscopy, a reliable and non-destructive method for analyzing cell wall architecture [42], revealed significant changes in the chemical structure of the pollen tube wall in the presence of PBA. Specifically, the abundance of de-esterified pectin and protein increased as a consequence of the PBA treatment, which was consistent with the immunolabeling results.

## Conclusions

In summary, we found that PBA treatment of apple pollen tubes promotes extracellular Ca^2+^ influx, elevates tip focused [Ca^2+^]c and eliminates the [Ca^2+^]c gradient, all or any of which may alter actin filament organization and cell wall composition, leading to perturbed tip growth. We conclude that boron is indispensable for maintenance of the typical tip-focused [Ca^2+^]c gradient, partially through extracellular Ca^2+^ influx. Our data also show that boron is important for the assembly and/or organization of the pollen tube actin cytoskeleton and some cell wall components, such as pectins and AGPs, especially in the apical region. This study provides new insights into boron function in the pollen tube polarized tip growth.

## Materials and Methods

### Plant material and culture

Ripe pollen grains were collected from *Malus domestica* trees in Henan Province (The north latitude 31°23′-36°22′, east longitude 110°21′-116°39′) on April 10, 2014. The collected pollen grains were place at room for several days for dehydration and then stored in vials at -20°0 until use.

The basic medium for pollen culture was composed by 20% (w/v) sucrose and 0.01% CaCl_2_, pH 6.8. Different concentrations of phenylboronic acid (C_6_H_5_B(OH)_2_)(purum, ≥97.0%, Sigma, St. Louis, MO, USA) were added to the culture medium in the beginning and shaken with 100 rpm at 30°0 in the dark.

Pollen germination rates were calculated according to Dafni [43] under a BX51 microscope with a CoolSNAP HQ CCD camera (Photometrics) after 2 h of incubation. Pollen tube length was measured using the Image-Pro Plus 6.0 software (http://www.mediacy.com/) after 2 h of incubation. All experiments were performed in triplicate and 150 pollen tubes were measured in each experiment.

### Measurement of extracellular Ca^2+^ influx

Method of Yu et al. [44] was employed to measure net Ca^2+^ fluxes in the Younger USA (Xuyue Beijing) NMT Service Center by using a Non-invasive Micro-test Technique (NMT-YG-100, Younger USALLC, Amherst, MA01002, USA) with the ASET 2.0 (Sciencewares, Falmouth, MA 02540, USA) and the iFluxes 1.0 (Younger USA, LLC, Amherst, MA 01002, USA) software packages. The obtained data were analyzed using Excel (Microsoft) according to Yu et al. [44].

### Fluo-3/AM loading to label [Ca^2+^]c

Fluo-3/AM ester (Sigma, St. Louis, MO, USA) was loaded into pollen tubes at low temperatures in the dark at a final concentration of 10 μM as previously described [45]. After 2 h of incubation, the pollen tubes were washed with culture medium several times and placed at room temperature for 1 h. The samples were imaged with a Leica TCS SP5 confocal laser scanning microscope (CLSM) (Leica Co., Germany), with excitation and emission wavelengths of 488 nm and 515 nm, respectively.

### Fluorescence localization of actin filaments

Fluorescence localization of actin filaments was manipulated as previously described [11] with some modifications. Pollen tubes were fixed, treated by 1% cellulose R-10 and 1% pectinase and 1% Triton X-100. Then the pollen tubes incubated in 0.2 μM Alexa Fluor 488 phalloidin (Invitrogen, USA) in PBS (pH 6.9) buffer for 2 h in the dark. Finally, the samples were washed and observed under a Leica TCS SP5 CLSM with excitation and emission wavelengths of 488 nm and 515 nm respectively.

### Localization of pollen tube wall components

Pectins and AGPs of the pollen tube wall was labeled as described by Chen et al. [26]. Labeling of cellulose with Calcofluor white (fluorescent brightener 28) was carried out as described by Lazzaro et al. [46]. 0.05% aniline blue was used to label callose according to [11]. The stained pollen tubes were observed and photographed under the microscope or CLSM mentioned above. Image-Pro Plus 6.0 software (http://www.mediacy.com/) was used to calculate the relative intensity of the fluorescent signal from cellulose, callose, de-esterified pectin, esterified pectin and arabinogalactan proteins respectively according to Chebli et al. [47].

Fourier-transform infrared analysis (FTIR) of pollen tube wall components was carried out according to Hao et al. [11].

## Supporting Information

S1 Fig Effects of PBA at different concentrations on *Malus domestica* pollen tubes. Scar bar = 50 μm. (A) Pollen tubes treated by 0.1 mM PBA. (B) Pollen tubes treated with 0.3 mM PBA. (C) Pollen tubes treated with 0.6 mM PBA. (D) Pollen tubes treated with 0.7 mM PBA.
(TIF)
