# Supplementary figures and images for: Addition of Phenylboronic Acid to Malus domestica Pollen Tubes Alters Calcium Dynamics, Disrupts Actin Filaments and Affects Cell Wall Architecture
Source: PLoS One. 2016 Feb 17;11(2):e0149232. doi: 10.1371/journal.pone.0149232 (PMC4757038; doi:10.1371/journal.pone.0149232)

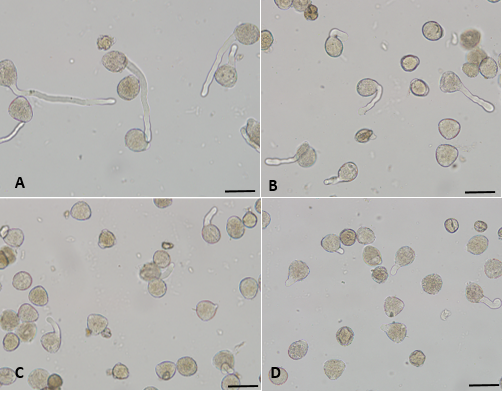

Supplement: S1 Fig — Scar bar = 50 μm. (A) Pollen tubes treated by 0.1 mM PBA. (B) Pollen tubes treated with 0.3 mM PBA. (C) Pollen tubes treated with 0.6 mM PBA. (D) Pollen tubes treated with 0.7 mM PBA. (TIF) [file pone.0149232.s001.tif]
